# Remodeling of retrotransposon elements during epigenetic induction of adult visual cortical plasticity by HDAC inhibitors

**DOI:** 10.1186/s13072-015-0043-3

**Published:** 2015-12-14

**Authors:** Andreas Lennartsson, Erik Arner, Michela Fagiolini, Alka Saxena, Robin Andersson, Hazuki Takahashi, Yukihiko Noro, Judy Sng, Albin Sandelin, Takao K. Hensch, Piero Carninci

**Affiliations:** Department of Biosciences and Nutrition, NOVUM, Karolinska Institutet, Stockholm, Sweden; Division of Genomic Technologies, RIKEN Center for Life Science Technologies, RIKEN Yokohama Institute, 1-7-22 Suehiro-cho, Tsurumi-ku, Yokohama, Kanagawa 230-0045 Japan; Lab for Neuronal Circuit Development, RIKEN Brain Science Institute, 2-1 Hirosawa, Wako-shi, Saitama, 351-0198 Japan; F. M. Kirby Neurobiology Center, Boston Children’s Hospital, Harvard Medical School, Boston, MA 02115 USA; Department of Biology and BRIC, The Bioinformatics Centre, University of Copenhagen, Copenhagen, Denmark; Department of Pharmacology, National University of Singapore, 10 Medical Drive 05-34, Singapore, Singapore; Department of Molecular and Cellular Biology, Center for Brain Science, Harvard University, 52 Oxford Street, Cambridge, MA 02138 USA; Genome Science Lab, RIKEN, Hirosawa, Wako-shi, Saitama 351-0198 Japan

**Keywords:** Visual cortex plasticity, DHSS, Chromatin, Retrotransposon elements, HDAC inhibitors, Enhancers

## Abstract

**Background:**

The capacity for plasticity in the adult brain is limited by the anatomical traces laid down during early postnatal life. Removing certain molecular brakes, such as histone deacetylases (HDACs), has proven to be effective in recapitulating juvenile plasticity in the mature visual cortex (V1). We investigated the chromatin structure and transcriptional control by genome-wide sequencing of DNase I hypersensitive sites (DHSS) and cap analysis of gene expression (CAGE) libraries after HDAC inhibition by valproic acid (VPA) in adult V1.

**Results:**

We found that VPA reliably reactivates the critical period plasticity and induces a dramatic change of chromatin organization in V1 yielding significantly greater accessibility distant from promoters, including at enhancer regions. VPA also induces nucleosome eviction specifically from retrotransposon (in particular SINE) elements. The transiently accessible SINE elements overlap with transcription factor-binding sites of the Fox family. Mapping of transcription start site activity using CAGE revealed transcription of epigenetic and neural plasticity-regulating genes following VPA treatment, which may help to re-program the genomic landscape and reactivate plasticity in the adult cortex.

**Conclusions:**

Treatment with HDAC inhibitors increases accessibility to enhancers and repetitive elements underlying brain-specific gene expression and reactivation of visual cortical plasticity.

**Electronic supplementary material:**

The online version of this article (doi:10.1186/s13072-015-0043-3) contains supplementary material, which is available to authorized users.

## Background

Patterns of neural activity shape the circuits that process sensory information during early windows of brain development [1]. Such critical periods of plasticity have been identified across modalities, including tonotopic map refinement in auditory cortex, barrel representation of whiskers in rodent somatosensory cortex, human language acquisition in Broca’s area and spatial acuity in the developing visual system [1–3]. Increased insight into how critical periods are regulated offers the potential to develop novel therapies for various neurological disorders or brain damage later in life. Recently, specific molecular players have been identified which control critical period timing in the visual cortex [1, 4, 5, 6]. While the rewiring capacity of the adult brain is known to be rather limited, the removal of certain molecular ‘brakes’ can be effective in recapitulating juvenile plasticity [7, 8].

Chromatin modulating factors in particular have been suggested to function as plasticity regulators. The class I histone deacetylase, HDAC2 inhibits memory formation and synaptic plasticity in the hippocampus [9], and the class II HDAC, HDAC5 is shown to be involved in depression [10]. Additionally, epigenetic mechanisms contribute to plasticity regulation and memory in the aging hippocampus [11]. Notably, acute pharmacological manipulation with HDAC inhibitors (HDACi) such as valproic acid (VPA) or trichostatin A (TSA) has been found to enable juvenile forms of cortical plasticity in adult rodents [12–14] and humans acquiring absolute pitch [15].

Classically acting as a potent inhibitor of GABA clearance, VPA has been a first-line drug treatment for epilepsy and manic disorder. More recently, VPA has been shown to be an HDACi as effective as, sodium butyrate, to induce epigenetic changes that promote recovery of visual acuity [13]. Indeed, its capacity as an HDACi has produced very promising therapeutic results in clinical trials for cancer, inflammatory diseases and central nervous system disorders [16–18]. At the same time, reopening of plastic brain states during cancer treatment may yield unwanted side effects [19]. A deeper mechanistic understanding is warranted whether HDAC inhibition by VPA induces local site-specific or genome-wide chromatin structure modulations in the brain.

Here, we show how HDAC inhibition by VPA influences chromatin structure and transcriptional control at a genome-wide level in a complex tissue such as the adult neocortex. We analyzed changes in genome-wide DNase I hypersensitivity sites (DHSS) of the visual cortex after either VPA or vehicle (Veh) treatment. Genome-wide studies of DHSS have been successfully performed in cell lines and primary suspension cells to accurately map regulatory regions in the genome [20–23]. In this study, we used DNase I to digest accessible DNA that is not protected by nucleosomes and compact chromatin formation. This yields the DHSS that correspond to nucleosome-free regions (NFR) and signature accessible regulatory regions, such as transcription start sites (TSSs), promoters, enhancers, silencers, insulators and locus control regions [21–25]. To understand transcriptional control, we constructed and sequenced cap analysis of gene expression (CAGE) libraries which precisely map the TSS location and estimate mRNA levels from VPA- and Veh-treated mice [26–29]. CAGE technology captures the cap structure in the 5′-end of mRNAs before they are processed and sequenced, resulting in genome-wide 5′-end transcript signatures to obtain precise TSS corresponding to the expression levels [30, 31]. We identified potential novel plasticity regulatory mechanisms, involving increased accessibility of enhancers, retrotransposon elements and in particular, short interspersed nuclear elements (SINEs). These elements were found to overlap significantly with computationally determined Forkhead box (Fox)-binding sites. Interestingly, VPA treatment induces the transcription of chromatin and neural plasticity regulatory genes, including *Foxg1*.

## Results

### Valproic acid reinstates ocular dominance plasticity and improves visual acuity in the adult visual cortex

Occluding one eye during the critical period (CP) (but not in adulthood) results in a shift of cortical spiking response (ocular dominance) in favor of the open eye and an enduring blunted vision (amblyopia) through the deprived eye [1]. A preliminary study with TSA in adult rats reported a reduced amplitude visual-evoked potential (VEP) upon deprivation [12] and the same group showed that VPA restores behavioral acuity to normal levels in adult rats previously deprived through that eye [13]. However, whether such reopened plasticity reflects changes in the spiking activity of single neurons within primary visual cortex remains unknown.

**Fig. 1:**
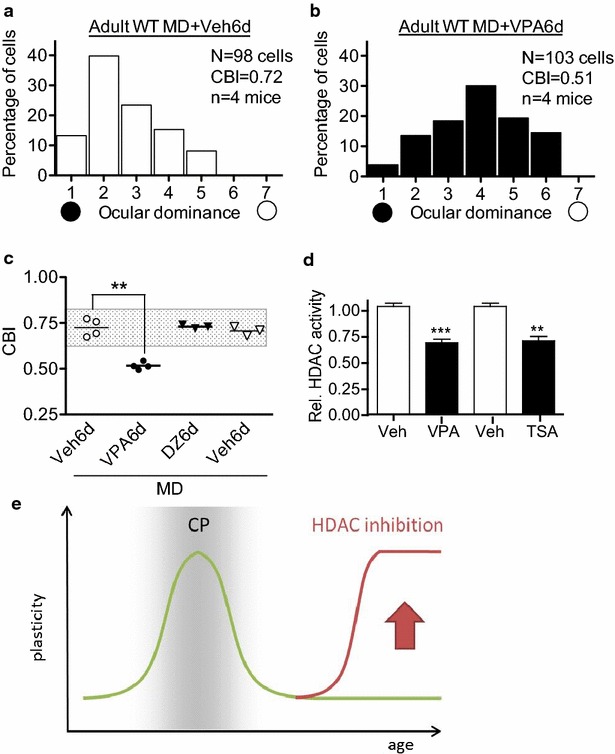
VPA reinstates ocular dominance plasticity in adult visual cortex. **a** The mouse visual system is naturally skewed toward the contralateral eye. Unlike during the CP [32], brief monocular deprivation (4d MD) fails to alter this ocular dominance of V1 neurons rated on a seven-point scale (*white bars*). **b** Upon VPA administration (6d) prior to and concurrent with adult MD, responsiveness once again can shift in favor of the open eye (*black bars*; *n* = 98 and 103 cells from *n* = 4 mice per group, CBI = 0.72 and 0.51, respectively; *p* < 0.001, *χ* ^2^ test). **c** Significant CBI reduction by MD occurs only with VPA (*closed circles*, *p* = 0.0002, unpaired two tailed *t* test versus vehicle, *open circles*). Enhancing inhibition, an alternative consequence of VPA treatment [33], is insufficient to induce adult plasticity, as benzodiazepine agonists (DZ, *closed triangles*; Veh, *open triangles*) fail to reduce CBI. **d** Reduction of histone deacetylase (HDAC) activity within 2 h of either valproic acid (VPA) or trichostatin A (TSA) administration. HDAC activity normalized to vehicle (***p = 0.0004, **p = 0.0024; unpaired two-tailed *t* test, *n* = 3 per group). **e** Treatment with HDACi re-activates the critical period in adult mice

We administered VPA (200 mg/kg, i.p. every 12 h) for 2 days prior to and concomitant with monocular deprivation (>4 days) of adult C57Bl/6J mice (P60) then performed single-unit electrophysiological recordings in vivo. Strikingly, VPA acutely restored a full ocular dominance shift comparable to that observed normally during a CP. Spiking activity in the mouse visual system is naturally skewed toward the contralateral eye, which is unaltered by sensory deprivation at this age (Fig. 1a, vehicle, upper panel, white bars). Monocular deprivation in the presence of VPA instead induced a shift toward the open-eye input as rated on a seven-point scale of neuronal responsiveness (Fig. 1b, VPA, lower panel, black bars).

The contralateral bias index (CBI) of individual animals captures the robustness of VPA-induced adult plasticity (Fig. 1c, VPA, closed circles). Since VPA is a known GABA transaminase inhibitor that would enhance endogenous inhibitory tone [33], we also tested whether directly enhancing GABA signaling with benzodiazepines might reopen adult plasticity (Fig. 1c, DZ, closed triangles). As expected [34], further strengthening inhibition after the CP produced no reduction of CBI, whereas VPA was totally effective. Moreover, HDAC activity in visual cortex was reduced by greater than 30 %, identical to Trichostatin A (TSA just 2 h after systemic injection of VPA (Fig. 1d).

Taken together, we confirmed the induction of a second period of plasticity in the adult mouse visual cortex by VPA (Fig. 1e), which is likely to reflect its action as an HDACi rather than a dampening of excitability.

### DHSSs in visual cortex are enriched in TSSs

To obtain a comprehensive view of how VPA treatment might change chromatin structure, we constructed DHSS libraries from VPA- (200 mg/kg, i.p.) and vehicle (saline)-injected mice. Libraries were prepared 120 min after single injection, consistent with a recent report that HDACi induces a transient increase of histone acetylation (75–150 min after injection) in brain tissues [35].

TSSs have previously been shown to be nucleosome free and correspond to DHSSs [22, 25, 36]. As expected, DHSS correlated better with highly expressed TSSs than those with low expression (Additional file 1: Figure S1a, b), reflecting that transcription levels associate with accessible promoter regions. Furthermore, there was a marked difference in enrichment between promoters with a well-defined (single peak) TSSs and promoters with multiple, widely spread (broad) TSSs, the latter being more highly enriched for DHSS tags (Additional file 1: Figure S1c, d).

**Fig. 2:**
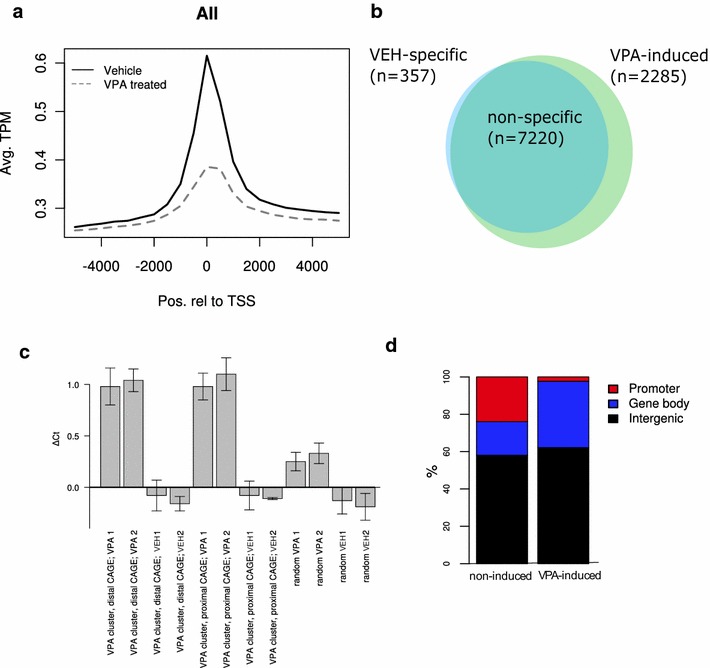
Distribution of DHSS in VPA- or vehicle-treated visual cortex. **a** Average DHSS tag enrichment around CAGE promoters from a previous study [31] from vehicle (Veh, *solid line*) and VPA-treated visual cortex (*dotted line*). Average TPM (tags per million) are computed in 500 bp bins centered around the TSS. Promoters located on chrM are excluded, as are promoters containing a bin with average TPM higher than 10 times the average TPM for that bin across all promoters. **b** *Venn diagram* showing the distribution of DHSS tags. **c** Validation of DHSS by qPCR. DNase I digested DNA from VPA- or vehicle-treated visual cortex were amplified with primer pairs annealing within induced DHSS proximal or distal to CAGE TSS clusters, or randomly in the genome. Data from two biological replicates for each treatment condition is shown (VPA 1 and 2, Veh 1 and 2). *Error bars* are standard deviations. **d** Genomic distribution of DHSS clusters with respect to RefSeq gene models

Strikingly, the enrichment of DHSS tags in TSSs was considerably stronger in the vehicle-treated sample compared to the VPA-treated sample (Fig. 2a). We hypothesize that this could be either a consequence of TSSs becoming less accessible after VPA treatment, or more likely, that the treatment induces a general increased genome availability occurring mainly outside of TSSs. The latter would lead to a diluted ratio of DHSS tags originating from TSSs in the VPA-treated sample.

### VPA treatment increases DNA accessibility in regions distant from promoters, including enhancers

To examine the extent to which VPA treatment induced and/or expunged sites of accessible chromatin in visual cortex, we clustered the DHSS tags and investigated their spatial location and specificity to either condition. Using a conservative threshold, 9862 clusters were identified (Additional file 2: Table S1), containing tags from VPA and/or vehicle-treated mice. Further analysis identified 2285 clusters with a significantly (one-tailed Fisher’s exact test, Benjamini-Hochberg 5 % FDR) higher number of tags from the VPA sample (from here on referred to as ‘VPA-induced DHSSs’). Conversely, only 357 clusters had a significantly higher number of tags from the vehicle condition. The remaining 7220 clusters contained tags from both conditions in similar proportion (Fig. 2b). This suggested that VPA treatment generally added sites of genome availability, rather than remove them. The 7577 clusters (357 specific to the vehicle condition and 7220 not specific to any condition) are from here on referred to as ‘non-induced DHSSs’.

Another set of mice in duplicate was injected with VPA or vehicle to confirm the DHSS libraries, and the appropriateness of the clustering method. The DNase I-digested chromatin was analyzed with a qPCR assay [37]. The VPA-induced DHSSs identified in the DHSS library, had an increased Ct value in the VPA-treated mice as compared to vehicle-treated mice, implying an enhanced sensitivity to DNase I (*p* value = 6.10956e−38) (Fig. 2c). This indicated our DHSS libraries and clustering method correctly identified true sites of accessible chromatin. We further designed primers at eight random locations outside of identified clusters. The changes in Ct value were approximately two- to threefold higher in mapped DHSSs compared to random sites (Fig. 2c). The fact that randomly picked locations had slightly higher Ct values in the VPA sample (*p* value = 1.1e−11) compared to vehicle may reflect a general effect on chromatin accessibility after VPA treatment. Together, these results suggest that VPA treatment gives rise to an increase in specific, and to a lesser extent general chromatin accessibility, and that our DHSS analysis identifies the chromatin regions that are more open and available.

Active enhancers bi-directionally transcribe short RNAs that can be analyzed with CAGE. Recently, this was used to map active enhancers in the FANTOM5 panel of tissues and primary cell types [38]. As expected, the DHSSs overlapped significantly (*p* = 8.7e−8, Binomial test) with enhancers (1.5 % of non-induced DHSS, 0.9 % of VPA-induced DHSSs). Moreover, DHSSs were found to be significantly closer to enhancers than expected by chance, *p* = 1.8e−5 (Kolmogorov–Smirnov test).

Further examination of the DHSS clusters demonstrated that the VPA-induced DHSSs have different characteristics compared to non-induced DHSSs. Genome-wide distribution of the respective DHSSs compared to RefSeq gene models [39] revealed an enrichment of induced DHSSs in gene bodies and not in known promoter regions compared to non-induced DHSS (Fig. 2d). Overall, these cluster analyses suggest that VPA treatment induces DHSSs rather than expunging them (Fig. 2b), and that induced regions are mainly located outside of TSSs (Fig. 2a, d).

### VPA induces brain-specific transcription distal to induced DHSSs

**Fig. 3:**
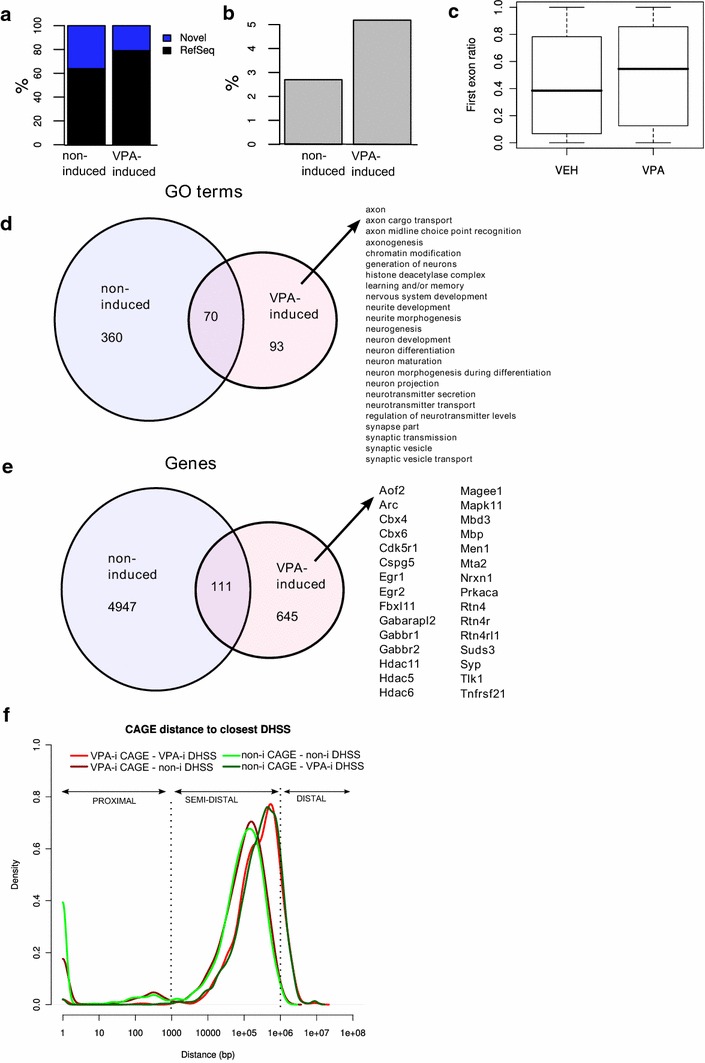
VPA-induced expression measured with CAGE. **a** Overlap between VPA-induced and non-induced TSS clusters (see main text for cluster definition) and RefSeq promoter regions. Promoter regions were defined as the region starting from 1 kb upstream of the RefSeq TSS and ending at the start of the coding region. **b** Overlap between VPA- and non-induced TSS clusters and previously identified CAGE promoters preferentially expressed in visual or somatosensory cortex. **c** Distributions (*box plots*) of the ratio of CAGE tags mapping to the first exon in the VPA- and non-induced samples). Ratios were computed by dividing the number of CAGE tags mapping to the first exon with the number of tags mapping anywhere along the length of the whole gene (including introns) in all genes with at least 10 CAGE tags mapping to the gene. **d** Selected gene ontology terms for RefSeq genes overlapping tss clusters (overlap definition as in A). VPA-induced and non-induced TSS clusters were analyzed separately using GOStat [42], the full list of significant terms is listed in Additional file 4: Table S3. **e** Overlap between RefSeq genes overlapping TSS clusters according to the definition in A, and a list of selected VPA induced genes associated with brain development and chromatin regulation. The intersecting genes have alternative promoters that respond differentially to VPA induction. The full list of genes is listed in Additional file 3: Table S2 along with TSS clusters. **f** Distributions (*density plots*) of the distance between VPA-induced (VPA-i) and non-induced (non-i) TSS clusters and the closest DHSS

To dissect how the perturbed epigenetic state affects transcription, CAGE was performed 2 h after VPA or vehicle treatment. Since the regions that were made accessible by VPA treatment mainly occurred outside of known promoter regions, we wanted to assess whether this was followed by transcription at novel promoters proximal to the induced DHSSs. Furthermore, since the induced sites were strongly enriched in gene bodies compared to the vehicle sample, another question was whether treatment resulted in exonic TSS [40, 41]. After sequencing and clustering, 9828 TSS clusters (Additional file 3: Table S2) were identified with 10 tpm (tags per million) or more in any of the two samples, out of which 1003 (14 %) were up-regulated in the VPA sample and 248 were downregulated (one-tailed Fisher’s exact test, Benjamini-Hochberg 5 % FDR). 6432 of the TSS clusters (65 %) were located in RefSeq [39] promoter regions (defined as the region starting 1 kb upstream of the RefSeq transcription start site reaching to the ATG), with the remaining 35 % constituting putative novel promoters. The VPA up-regulated TSS clusters (from here on referred to as ‘induced TSS clusters’) could be mapped to RefSeq TSS at a significantly higher degree (79 %, *p* < 2.2e−16, one-tailed Fisher’s exact test) than for active, but not induced TSS clusters (from here on referred to as ‘non-induced TSS clusters’) after treatment (64 %, Fig. 3a).

DHSS tags in both induced and non-induced samples were enriched at TSS clusters, although with a significantly stronger enrichment in the non-induced sample. Many of the TSS clusters (70 %) overlapped with previously defined deepCAGE promoters [31]. Moreover, induced TSS clusters overlapped previously identified clusters preferentially expressed in either visual or somatosensory cortex to a significantly higher degree than non-induced TSS clusters (5.2 vs. 2.7 %, *p* = 3.4e−5, one-tailed Fisher’s exact test, Fig. 3b). Furthermore, VPA-treated visual cortex demonstrated an increased ratio of CAGE TSS in exon 1 compared to other exons (*p* < 2.2e−16, two-tailed *t* test, Fig. 3c), indicating that VPA resulted in reduced scattered transcription within genes. Despite induction of major global histone acetylation and putative enhanced DNA accessibility, the induced promoters showed high site specificity with regard to TSSs.

It has previously been reported that VPA treatment induces an epigenetic configuration that resembles a pre-plastic state, with high histone acetylation, which leads to a renewed CP in visual cortex, [12, 43]. Consequently, gene ontology (GO) analysis of the induced genes revealed induction of several classes of GO terms, including “chromatin modulation” and “histone deacetylase complex” (Fig. 3d). Additionally, many brain-related processes were induced, such as learning and/or memory, neurogenesis, neuron development and neuron projection (partial list of enriched terms in Fig. 3d and full list Additional file 4: Table S3), indicating that VPA treatment induces plasticity-related biological processes, which are dormant in the adult visual cortex. VPA treatment induced expression of TSS clusters of 645 genes annotated as described above, as well as alternative TSS clusters in an additional 111 genes already expressed in untreated visual cortex (Fig. 3e). As indicated by the GO terms, the gene list includes epigenetic regulators, such as *Hdac11, Hdac6, Hdac5,Fbxl11,Mbd3*, *Tlk1, Mll3, Cbx4 and Cbx6* as well as several genes implicated in brain development and plasticity: *Arc, Egr1/2,Suds3, Aof2, Mta2, Syp* and *Magee1*. Notable among these were the Nogo-receptor (*Rtn4r*) signaling pathway, which binds to extracellular inhibitors of neurite outgrowth, such as chondroitin sulfate proteoglycans (*Cspg5*) and myelin-related factors (*Mbp, Rtn4*) [44–46].

No association between induced DHSS and TSS was detected. But, investigation of the spatial distribution of active promoters represented by our TSS clusters, compared to the genomic locations of detected DHSS clusters, revealed that a majority of induced (89 %) as well as non-induced (87 %) TSS clusters were located between 1 kb and 1 Mb (semi-distal) from the closest non-induced DHSS (Fig. 3f). Similarly, the majority of TSS clusters (induced 88 %, non-induced 87 %) were located semi-distal to the closest induced DHSS. However, a striking difference was observed when considering DHSS proximal (within 1 kb) or distal (further than 1 Mb) to TSS clusters. A substantial number of clusters (induced TSS clusters 11 %, non-induced TSS clusters 14 %) were located proximal to the closest non-induced DHSSs, whereas only few TSS clusters (induced 1 %, non-induced 1 %) were located proximal to VPA-induced DHSSs. Conversely, very few promoters (induced 0.1 %, non-induced 0.4 %) were located distal to non-induced DHSSs, while a substantial number of TSS clusters (induced 11 %, non-induced 13 %) were located distal to induced DHSSs. These results suggest that non-induced DHSSs may be involved in proximal, positive regulation of non-induced as well as induced TSS clusters, whereas the induced DHSS may represent mainly distal regulation or repression.

Thus, a seemingly unspecific epigenetic perturbation (VPA) causes removal of nucleosomes at regions distal to active TSS clusters, which may be the consequence of a repressive effect, and induces a tissue-specific mRNA expression mainly from known promoter regions.

### VPA-induced DHSSs overlap with SINE and putative Fox-binding sites

**Fig. 4:**
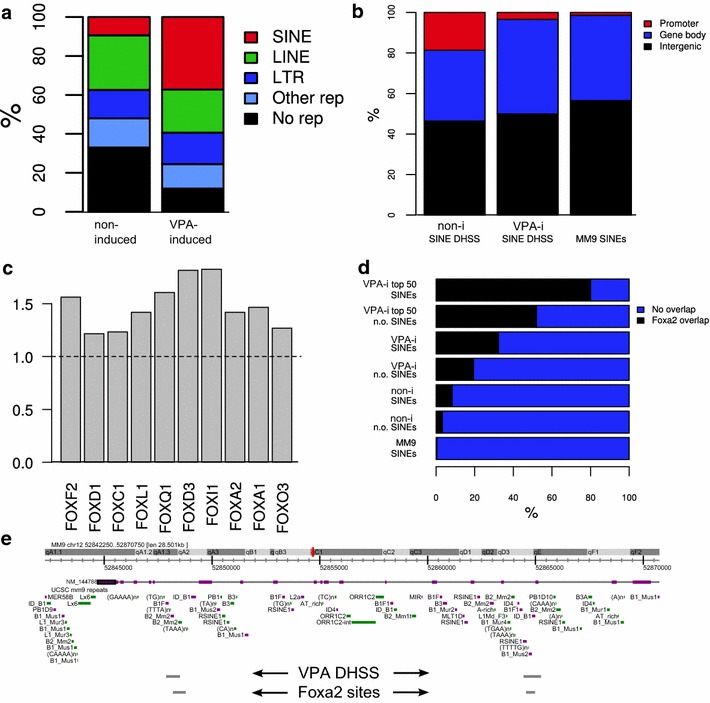
VPA induces DHSS at retrotransposon elements. **a** Genomic distribution of DHSS with respect to repeat regions (defined by RepeatMasker [http://www.repeatmasker.org]). In cases where the same DHSS overlapped several repeats, the overlap with the greatest length was chosen. **b** Genomic distribution of SINEs overlapping non-induced (non-i) DHSS (*leftmost bar*), VPA-induced (VPA-i) DHSS (*mid bar*), and all SINEs (*rightmost bar*), with respect to RefSeq gene models. **c** Over-representation of forkhead motifs in induced SINE DHSS compared to all other DHSS. For each JASPAR forkhead motif, the number of matches (obtained using the TFBS toolkit) with at least 80 % similarity were compared between the two groups using one-tailed Wilcoxon rank-sum test. In all cases, *p* < 1.5e−16. **d** Overlap between SINEs and Foxa2 binding sites in mouse liver. “non-induced (non-i)/VPA-induced (VPA-i) SINEs” indicates DHSS clusters that overlap SINEs, and “non-induced/induced n.o. SINEs” indicates clusters that do not overlap SINEs. “Top 50 VPA SINEs/n.o. SINEs” indicates the 50 VPA DHSS clusters with highest DHSS tag count in the VPA-i sample, which do or do not overlap SINEs, respectively. **e** Genome browser view of the 3′ end of the Hectd1 gene (NM_144788), indicating an overlap between VPA DHSS, SINEs and Foxa2 binding sites

A detailed analysis of the genomic distribution of the DHSS clusters revealed that a large fraction of the induced DHSS overlaps with retrotransposon elements—out of the induced DHSS, 88 % were located in retrotransposon elements, compared to 67 % of the non-induced DHSS. Specifically, a strong enrichment in SINE elements was observed, with 851 induced DHSS (37 %) having their longest overlap with a SINE element compared to 9 % of non-induced DHSS (Fig. 4a). Considering all such DHSS (including those where a longer part of the region overlapped another repeat type), in total 1209 induced DHSSs (53 %) overlapped a SINE element. Upon further analysis of the induced DHSSs, SINE elements showed a specific over-representation in B1 elements (corresponding to human Alu elements) (Additional file 1: Figure S1e). The distribution of VPA-induced SINE DHSSs closely resembled the general genomic distribution of SINE elements, with a minority located in promoter regions and most located in the gene bodies or intergenic regions (Fig. 4b). In contrast, non-induced SINE DHSSs had a stronger tendency towards promoter regions.

The similarity in pattern between induced DHSS SINE elements and the genomic SINE distribution suggests that SINEs in general are not targeted by VPA treatment. However, further analysis revealed a strong deviation from general genomic SINE patterns. Scanning the DHSS clusters for putative transcription factor-binding sites (using the TFBS toolkit [47] and JASPAR [48] collection of DNA-binding matrices), FOX family member-binding sites were identified as being significantly over-represented in VPA-induced SINEs (Fig. 4c) as compared to all other identified DHSS clusters.

The FOX family members have a conserved DNA-binding domain and bind to similar DNA sequences [49–52]. Comparing our induced DHSSs to publicly available Foxa2-binding sites identified by ChIP-seq in mouse liver [53], a dramatically strong overlap was observed (Fig. 4d). Out of 1209 induced DHSSs overlapping with SINEs, 391 (32 %) overlapped Foxa2 sites compared to 0.5 % out of the approximately 1.5 million annotated SINEs in the mouse genome overlapping these Foxa2 sites (*p* < 2.2e−16, Fisher’s exact test). VPA-induced DHSSs not overlapping SINEs were also enriched in Foxa2-binding sites, but to a significantly (*p* = 2.1e−12) lower degree.

Among the top 50 most sequenced induced DHSSs overlapping SINEs, 80 % had embedded Foxa2-binding sites (Fig. 4d). Non-induced DHSSs overlapping SINEs were also enriched for Foxa2-binding sites, but overlapped these sites to a substantially lower degree than the induced DHSSs (3 vs 1 % non-induced DHSSs not overlapping SINEs, Fig. 4d). We conclude that VPA treatment induces a highly specific response, targeting SINE elements harboring forkhead box-binding sites (Fig. 4e), and that these proteins may play an important role in the VPA response.

*Foxa2* is primarily expressed in liver, pancreas and lung [54], so consequently, no CAGE TSS clusters were present in the Foxa2 gene from our visual cortex samples. However, several other forkhead proteins were expressed, including *Foxj1* and *Foxk1* in both untreated and VPA-treated visual cortex. Instead, *Foxg1* (also known as Brain Factor-1, BF-1) was significantly up-regulated in the VPA CAGE library (Additional file 5: Figure S2a). The induced *Foxg1* TSS cluster corresponds to a novel transcript (BC064449) described previously [55]. The Foxg1 consensus-binding site is very similar to the Foxa2-binding motif (Additional file 5: Figure S2b), and likely to bind to the same motif [56]. The up-regulation of *Foxg1* was verified with qPCR (Additional file 5: Figure S2c).

## Discussion

### Valproic acid reinstates ocular dominance plasticity in the adult visual cortex

Alterations in spike output from the primary visual cortex ultimately underlie the CP for visual acuity [32]. We first confirmed that VPA enables ocular dominance plasticity at the single neuron level within the adult visual cortex of mice. This corroborates earlier findings in adult rats using local field potentials (VEP) with TSA, or whole animal behavior to assess acuity recovery with VPA [12, 13, 32]. Importantly, VPA is just as potent as TSA in reducing HDAC activity. Other confounding actions of VPA, such as dampening excitability, are unlikely to have induced adult plasticity, since direct GABA enhancement with benzodiazepines at this age failed to do so (Fig. 1c). We, therefore, explored chromatin reorganization by VPA in visual cortex in detail.

### VPA-induced DHSS correspond to SINEs and Fox-binding sites

Recently, the importance of retrotransposon elements including SINEs for brain development has been demonstrated [57, 58]. Accordingly, we find that SINEs are correlated with CP reactivation in visual cortex and these have embedded Fox-binding sites (Fig. 4c, d). Fox proteins bind DNA with high affinity, also when it is wrapped around nucleosomes, disrupting and opening up compact chromatin structures with nucleosome re-arrangement [51, 59, 60, 61]. FoxA1-binding does not induce transcription, but it has a profound effect on nucleosome organization and creates DHSS [62, 63]. FoxA1 has been shown to be recruited mainly to enhancers with only approximately 2 % of binding sites located at proximal promoters [63, 64], creating a nucleosome-free region while stabilizing the surrounding nucleosomes [64], and stable DHSS at enhancers.

Notably, FoxA1 is on the same evolutionary branch as FoxG1 [62], suggesting FoxG1 protein may create DHSSs by nucleosome remodeling in a similar manner in neurons. SINEs have been shown to be over-represented within gene-rich regions, while LINEs and LTRs are under-represented [65]. Binding sites for several other transcription factors are proposed to be embedded in distinct families of retrotransposon elements [66–68]. We recently showed that embryonic stem cell (ESC) factors such as NANOG, SOX2 and OCT4 bind in LTR-associated regulatory regions in ESC [69]. SINE Alu/B1 elements contain bindings sites for transcription factors that are involved in cell development [70, 71]. Ours is the first report to our knowledge that identifies SINEs as carriers of Fox-binding sites (Fig. 4c, d).

VPA incubation has previously been shown to induce both histone acetylation and H3K4 methylation [72, 73]. In vivo FoxA1 binding has been demonstrated to correlate with H3K4 mono and dimethylation of enhancer elements [64]. This is consistent with our observation that Fox-binding sites become accessible after VPA treatment. While some Fox-binding sites demonstrate tissue specificity, a significant proportion of sites are suggested to be shared between different cell types [74]. The strong overlap between VPA-induced SINE DHSS in visual cortex and Foxa2-binding sites in mouse liver observed here may, therefore, be an underestimation of Fox binding in the induced sites. Different Fox members can also be acetylated at several sites [75]. Acetylation of FoxA1 has been suggested to reduce its binding to DNA [76], while acetylation of FoxP3 has been shown to increase the affinity to DNA and stabilize the Fox proteins [75]. Consequently, treatment with HDAC inhibitors may affect the affinity to DNA and protein stability of Fox proteins, in addition to increasing the accessibility of FOX-binding sites.

The nucleosome-free SINE elements may help to re-program the genome organization in the nucleus. Transcription factor TFIIIC complex binds to B-boxes and is proposed to form chromosome-organizing clamp (COC) in fission yeast [77]. TFIIIC loci tether distant loci to the nuclear periphery, without RNA polymerase binding, and are suggested to regulate genome organization [77]. Also, in budding yeast Pol III independent TFIIIC loci are detected [78], indicating that it may be a conserved phenomenon. Interestingly, Alu repeats contain B-box sequences and thus represent a large fraction of potential TFIIIC-binding sites that may play a role in genome organization. The large pool of Alu repeats that becomes available after VPA treatment (Fig. 4a; Additional file 1: Figure S1e) may play an important role in re-programming neurons and glial cells by nuclear reorganization. However, TFIIIC binding and COCs remain to be demonstrated in VPA DHSSs. Repetitive elements not only serve as promoters, but may also be an important regulator of nuclear structure. Retro-transposition has recently been suggested to contribute to neuronal plasticity with a prevalence of neuronal genes [70, 79]. Retro-transposition also takes place in ESCs and suggested to be important for early development [80].

Expression of retrotransposon elements has been demonstrated to be cell and tissue specific [81]. However, we could not detect enhanced transcriptional activation of SINEs implied by the increased accessibility to SINEs. In recent studies, it has been shown that RNA, expressed from retrotransposon elements is predominantly in the nuclear fraction and not in the cytosolic fraction [82]. Deep transcriptome profiling of mammalian stem cells supports a regulatory role for retrotransposons in pluripotency maintenance and that CAGE transcripts of long non-coding RNA are primarily detected in nuclear RNA libraries [82]. It may, therefore, be due to technical limitations that we fail to detect transcription from the induced SINEs in our whole cell RNA libraries. Moreover, SINEs have been reported to be transcribed in the sense direction, by RNA polymerase III and do not get 5′-capped in this process [83, 84]. Therefore, it is possible that elevated SINE element transcription, if it occurs, could not be detected with CAGE in this study.

### VPA induces global chromatin modification but brain-specific gene expression

Different retrotransposon elements may function as regulatory elements in different tissues [66, 68, 69, 81, 85, 86] and their roles are just starting to be deciphered. Recently, it was discovered that Alu insertions can function as gene promoters [51–53]. Interestingly, although many different types of retrotransposon elements are transcribed in a tissue [81], VPA induces availability specifically in SINEs. The distance between VPA DHSSs and expressed TSS clusters is usually from 10 kbp up to several Mbp long (Fig. 3f), indicating that the induced DHSSs are not mainly involved in proximal regulation of transcription. The induced DHSSs may, therefore, be involved in distal gene regulation, repression, or regulation of higher chromatin structure. This is in agreement with the majority of DHSSs previously being described as located distal from genes [20]. Indeed the induced DHSSs are enriched for enhancer regions. Incidentally, also other retrotransposon elements have been found to specifically act as cell-specific enhancers. In both human and mouse iPS and ESCs, a different class of retrotransposon elements mostly constituted by ERVK and ERV1 LTR elements has recently been found to produce cell-type restricted enhancers [69]. These elements in iPS/ESCs act as distal regulators involving transcription factors that are necessary for pluripotency. This finding reinforces the concept that specific retrotransposon elements can control specific cellular states or response, while further studies in other system may further broaden the role of retrotransposon elements in gene regulation. In contrast, a subset of promoters that became activated after VPA treatment has proximal DHSSs in both treated and untreated visual cortex. These TSS clusters may represent genes that are poised for expression upon specific signaling.

We detected a high specificity in up-regulation of visual cortex-related genes, despite the dramatic genome-wide change in the epigenetic landscape after HDACi injection. It has previously been shown that treatment with HDACi activates only a subset of promoters [35, 87, 88]. Thus, a seemingly unspecific drug results in a specific response. Genes primed with H3K4 trimethylation are suggested to be more easily acetylated, when treated with HDACi [35, 89], implying that genes may need to be poised for transcription to be activated by HDACi treatment. Lopez-Atalaya et al. have shown that the acetylation levels in hippocampus increase in gene bodies in active genes that are pre-marked with H3K4 trimethylation and H3K9/14 acetylation, and to some extent also regions distant (>10 kb) from any annotated gene, after TSA treatment [38]. These are the same genomic regions that were induced DHSSs located after VPA treatment (Fig. 1). In both our study and that of Lopez-Atalaya et al. [32], the induced histone acetylation/DHSS and gene expression are uncoupled and a portion of the induced sites located in enhancers [38]. However, in both studies expression of HDACs is induced as a response to treatment with HDACi (Fig. 3e). Among them is HDAC11, which has previous been shown to peak specifically during the ocular dominance critical period in visual cortex [90]. The tissue specificity of the induced sites may then reflect the relative abundance of regulatory transcription factors in visual cortex and hippocampus, respectively, since in both cases the sites are enriched for specific transcription factor-binding sites (Fig. 4c–e) [38].

Several plasticity and chromatin regulatory genes became up-regulated through VPA treatment. However, the induced DHSS were mainly not located in promoter regions, but at a long distance from TSS clusters (Fig. 3f). Cell-specific enhancer elements, identified by mapping p300 binding sites to the genome, have been shown to act at a very long distance from promoters [90]. Only a few percent of the p300 enhancers map within promoter regions and approximately 40–50 % lie 10–100 kbp away and 20–30 % further than 100 kbp distant from promoters [90]. The enhancer distribution resembled the induced DHSS locations (Fig. 3f), suggesting that the induced DHSS are enhancer regions. It has previously been shown that enhancers can function at >1 Mbp distant to their gene promoter target [91, 92]. Indeed both the non-induced and induced DHSS are significantly enriched for enhancer elements. Consequently, despite the distant location from TSS clusters, the induced DHSS may regulate the induced brain-specific gene expression.

Furthermore, the expression of Foxg1 is enhanced by VPA treatment (Additional file 5: Figure S2a, c). Foxg1 acts mainly as a transcriptional repressor and interacts with the co-repressors Groucho family and HDAC1 [93]. Interestingly, Foxg1 interacting partners TLE/Groucho were up-regulated in our CAGE VPA library (Additional file 3: Table S2). This finding indicates that Foxg1 may play an important role in re-establishing a juvenile plastic state in visual cortex, as Foxg1 is implicated to be involved in neurogenesis and neuronal stem cell self-renewal [94–96], radial versus horizontal cell migration [97] and suppresses early cortical cell fate [98]. Recently Foxg1 was suggested to cause a shift towards GABAeric neuron fate and alter the transcriptome network that is involved autism spectrum disorder [99]. Thus, further stress the importance of Foxg1 in the developing brain.

Notably, CP timing is exquisitely sensitive to the maturational state of GABA neurons [1], suggesting that transcriptomic changes induced by VPA in adulthood is acutely recapitulating a juvenile form of excitatory–inhibitory balance conducive to plasticity. The visual cortex is composed of many different cell types, both neurons and non-neurons, such as glia cells. It is likely that the different cell types will respond differently to the VPA treatment, making detailed analysis of a whole complex tissue like the visual cortex challenging. With rapid developments in single-cell technologies, it may soon be possible to dissect the correlation, response to HDACi and role in plasticity, in a cell-specific manner.

## Conclusions

We find that short-term VPA treatment causes a dramatic reorganization of chromatin structure: (a) genome-wide formation of DHSS that overlap enhancer regions. (b) A genome-wide enhanced accessibility to repetitive elements, in particular SINE elements that overlap with putative Fox-binding sites. (c) Induction of specific gene transcription, including neuronal and epigenetic regulatory genes.

## Methods

### Animals

Adult C57BL/6J mice (>postnatal day P56) were maintained on a 12-h light/dark (LD) cycle and had access to food and water ad libitum.

Drug administration for plasticity analysis: Valproic acid (VPA; 200mgkg-1, i.p; Sigma-Aldrich) was dissolved in sterile saline. Vehicle or VPA was i.p. injected twice a day. Diazepam (DZ; 2 mg ml^−1^ in 50 % propylene glycol/PBS, i.c.v; Sigma-Aldrich). Vehicle or DZ was injected daily into both lateral ventricles (1.5 μl per hemisphere and a total of 3 μl) for 2 days.

Monocular deprivation and drug administration: Prior to monocular deprivation (MD) experiments, VPA was injected into wild-type adult mice every 12 h for 2 days. After the 2nd day, eyelid margins were trimmed and sutured under halothane anesthesia for 4 days (brief MD). VPA was administered i.p. every 12 hourly over another 4 days. All recordings were obtained contralateral to the deprived eye and blind to drug treatment.

Drug administration for DHSS and CAGE analysis: Valproic acid (VPA; 200 mg kg^−1^, i.p; Sigma-Aldrich) was dissolved in sterile saline. Equivalent volume of vehicle solution (Veh) was injected into control animals. Visual cortex was excised 2 h after Veh or VPA treatment for RNA extraction using RNAeasy kit (Qiagen) or for DHSS assay. All animal protocols were approved by the Institutional Animal Care and Use Committee (IACUC) at the RIKEN Brain Science Institute, Japan.

### Electrophysiology

Electrophysiological recordings were performed under Nembutal (50 mg kg^−1^; Abbot)/chlorprothixene (0.2 mg; Sigma) anesthesia using standard techniques [34].

### HDAC activity

HDAC activity was measured using HDAC Fluorometric Assay/Drug Discovery kit (BIOMOL research Laboratories Inc.), according to manufacturer’s instructions.

### DHSS library production

Two visual cortices were resuspended in ice-cold resuspension buffer [50 mM Tris–HCL (pH 7.5), 0.8 M sucrose, 150 mMKCl, 5 mM Mg_2_Cl_2_]. The tissue was homogenized with a pellet pestle at the lowest speed for 20 s. Cells were lysed by incubating on ice for 10 min with 5 ml buffer A: 7.5 mM Tris–HCl (pH 7.4), 7.5 mM NaCl, 2.5 mM MgCl_2_, 30 mM KCl, 0.05 mM EGTA, 0.5 mM DTT, 125 µM PMSF, 160 mM sucrose, 0.5 % NP40, 0.5 mM spermidine and 0.1 M trehalose. The nuclei were washed in buffer A, without NP40, supplemented with 20 % glycerol and 0.3 M sucrose and resuspended in 200 μl buffer C: 10 mM Tris–HCl (pH 7.4), 15 mM NaCl, 3 mM MgCl_2_, 0.6 mM CaCl_2_, 0.5 mM DTT and 0.25 M sucrose. The digestion was carried out at 22 °C with 2 units DNase I for 15 min and RNase treated for 10 min at 37 °C. Addition of 200 μl stop buffer [100 mM NaCl, 100 mM EDTA (pH 8.0), 1 % SDS, 50 mM Tris–HCl (pH 8.0)] stopped the reaction. The digestion was controlled with Pulse Field electrophoresis.

After digestion, the sample was treated with proteinase K and DNA extracted with phenol/chloroform. The DNA was washed and purified on Microcon YM-30 columns. The DNase I digestion sites were mended with T4 DNA polymerase and 30 pmol biotinylated adaptor containing one Mme1 and one XbaI restriction site, was ligated to the mended cut sites. The unligated adaptors were removed using Microcon YM 100 column. The DNA was digested with MmeI and the adaptor-tag construct was purified with streptavidin-coated magnetic beads (Dynalbeads M280). While bound to the magnetic beads a second adaptor including an XbaI site was ligated to the constructs in ligation buffer supplemented with 0.5 M trehalose. After XbaI digestion, the DNA was ligated to Solexa bar-coded adaptors, amplified, purified on PAGE gels and sequenced on the Solexa platform.

### DHSS tag mapping and clustering

73006924 sequenced and extracted tags were mapped to the mouse genome (mm9) using Nexalign (described in [100]) allowing for at most one mismatch. 63 % (26777479 VPA tags and 19241564 vehicle tags) mapped uniquely and were retained for further analysis.

The genome was scanned in windows of 10 kbp size, and each was further scanned in 500 bp windows. Consecutive 10 kb windows overlapped by 400 bp, and consecutive 500 bp windows overlapped by 100 bp. Given length *L* of the big window after correction for repetitiveness (by considering the number of potential single mapping locations for 21-mers), the length *l* of the small window after the same correction, the number of tags *N* in the big window, the number of tags *n* in the small window, the expected number of tags *E*(*n*) and standard deviation *σ* were calculated using the binomial distribution with *p* = *l*/*L*. Simultaneously, in each small window, a random number *n*_rand_ was drawn from the same distribution, and *E*(*n*_rand_) and *σ*_rand_ were computed in the same way. This procedure was done separately for vehicle and VPA samples in each window. Subsequently, a combined normalized *z* score *z*_norm_ = (*n*_norm_ − *E*(*n*_norm_))/*σ*_norm_ was calculated, with *n*_norm_ = *n*_*veh*_ + *n*_VPA_ × *N*_tot,veh_/*N*_tot,VPA_, *E*(*n*_norm_) = *E*(*n*_veh_) + *E(n*_VPA_*)* × *N*_tot,veh_/*N*_tot,VPA_, and *σ*_norm_ = sqrt(*σ*_veh_^2^ + *σ*_VPA_^2^ × (*N*_tot,veh_/*N*_tot,VPA_)^2^). *N*_tot,veh_ and *N*_tot,VPA_ represent the total number of single mapping DHSS tags from the vehicle and VPA sample, respectively. Similarly, a *z* score *z*_rand_ was computed from *n*_rand_ and *σ*_rand_.

Finally, a cutoff *z*_cutoff_ was chosen based on the distributions of *z*_norm_ and *z*_rand_, to give a 5 % false discovery rate (FDR). *z*_cutoff_ = 5.5 fulfilled this criterion. Overlapping clusters with *z*_norm_ ≥ *z*_cutoff_ were merged, yielding 9862 clusters retained for analysis. For each of the 9862 clusters, a one-tailed Fisher’s exact test was performed to see whether it had a significantly greater amount of tags from the VPA condition. Clusters with FDR corrected *p* value (Benjamini-Hochberg) <0.05 were assigned as VPA induced.

### Validation of DHSS with qPCR

Real-time PCR was performed as previously described [21, 37]. Nuclei isolation and DNase I treatment were performed as in DHSS library production protocol. Eight primer pairs in each of the following categories were designed to anneal: to VPA-induced DHSS at non-TSS SINE DHSS, at TSS ± 2kbp or at random genomic sites. After DNase I digestion, amplicons could not be produced if the DNA region were open and available for DNase I digestion, which imply a higher Ct value than undigested DNA. Each PCR was performed in triplicate using DNA from VPA- or vehicle-treated visual cortex as template. One aliquot from each DNA sample was digested with DNase I and a corresponding aliquot was undigested. All qPCR were performed on 7500 Real-time PCR System (Applied Bioscience) using an SyBR green assay. The ΔCt values = Ct (digested DNA) − Ct (undigested DNA). Statistical evaluation was performed with two-tailed *t* test, assuming unequal variance.

### CAGE

CAGE was performed as previously described [30]. 2287755 CAGE tags were sequenced, extracted and mapped to the mouse genome (mm9) using Nexalign allowing for at most two mismatches. 62 % (431797 VPA tags, 1042302 vehicle tags) mapped to unique locations and were subjected to clustering.

Single linkage TSS clusters were constructed by merging overlapping CAGE tags as previously described [29, 31]. 9828 clusters with at least 10 tags per million (TPM) in either or both samples were retained for further analysis.

## Data availability

CAGE and DHSS sequencing data are available in DDBJ at the following accession number: DRA004104.

## Additional files

10.1186/s13072-015-0043-3 DHSS and transcription start sites. DHSS overlap more with highly expressed than low expressed genes in both VPA (A) and vehicle (B) treated animals. DHSS are enriched in broad promoters compared to single peak promoters in both VPA (C) and Veh (D) treated animals. (E) DHSS are enriched in the Alu/B1 subclass of SINE elements.
10.1186/s13072-015-0043-3 Table of identified DHSS.
10.1186/s13072-015-0043-3 Table of identified CAGE clusters with ≥10 tpm (tags per million).
10.1186/s13072-015-0043-3 Complete list of GO-terms.
10.1186/s13072-015-0043-3 VPA-induced FoxG1 expression with binding sites which overlap VPA-induced DHSS. (A) Genome browser view for the Foxg1 locus. CAGE tags are significantly upregulated in the VPA sample compared to vehicle, at a start site downstream of the canonical RefSeq TSS, corresponding to previously described transcript BC064449. (B) Comparison between the Foxg1 binding motif and the Foxa2 motif. (C) qPCR validation for induced FoxG1 expression after 2 h VPA treatment.

## Abbreviations

DHSS: DNase I hypersensitive sites
CAGE: Cap analysis of gene expression
VPA: Valproic acid
TSS: Transcription start sites
SINE: Short interspersed nuclear elements
HDACi: Histone deacetylase inhibitor
