# Supplementary figures and images for: Remodeling of retrotransposon elements during epigenetic induction of adult visual cortical plasticity by HDAC inhibitors
Source: Epigenetics Chromatin. 2015 Dec 14;8:55. doi: 10.1186/s13072-015-0043-3 (PMC4678690; doi:10.1186/s13072-015-0043-3)

**A****VPA treated**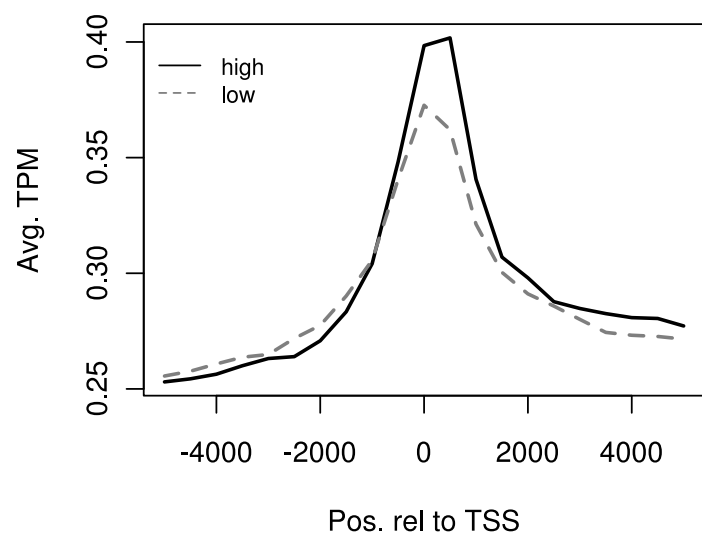**B****Vehicle**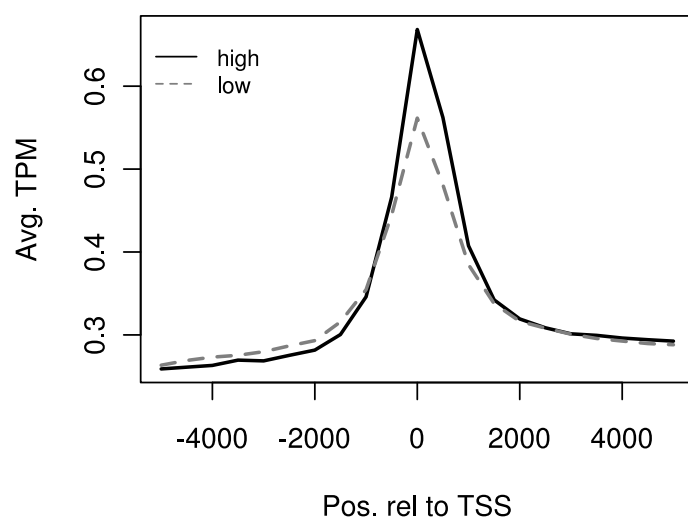**C****VPA treated**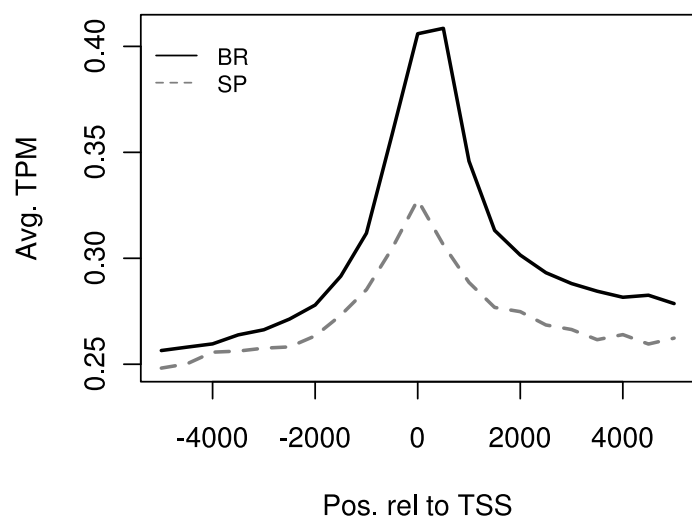**D****Vehicle**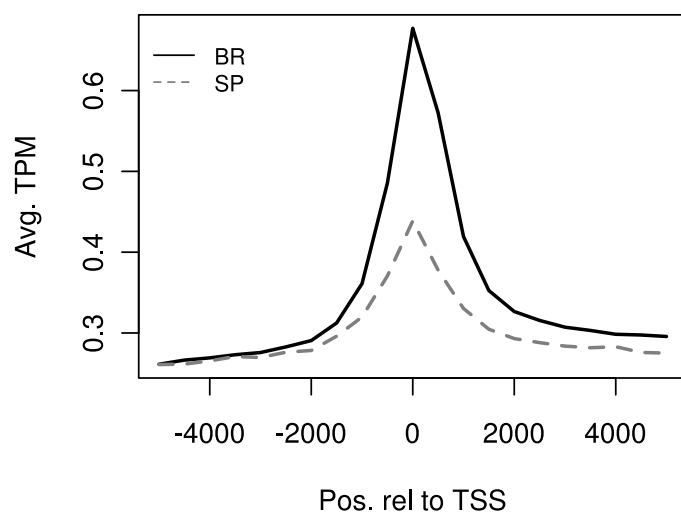**E**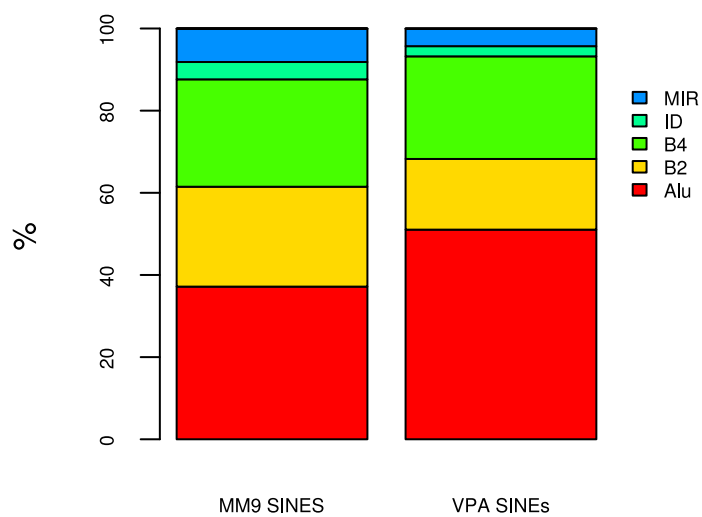

Supplement: Supplementary file 1 — 10.1186/s13072-015-0043-3 DHSS and transcription start sites. DHSS overlap more with highly expressed than low expressed genes in both VPA (A) and vehicle (B) treated animals. DHSS are enriched in broad promoters compared to single peak promoters in both VPA (C) and Veh (D) treated animals. (E) DHSS are enriched in the Alu/B1 subclass of SINE elements. [file 13072_2015_43_MOESM1_ESM.pdf]

A

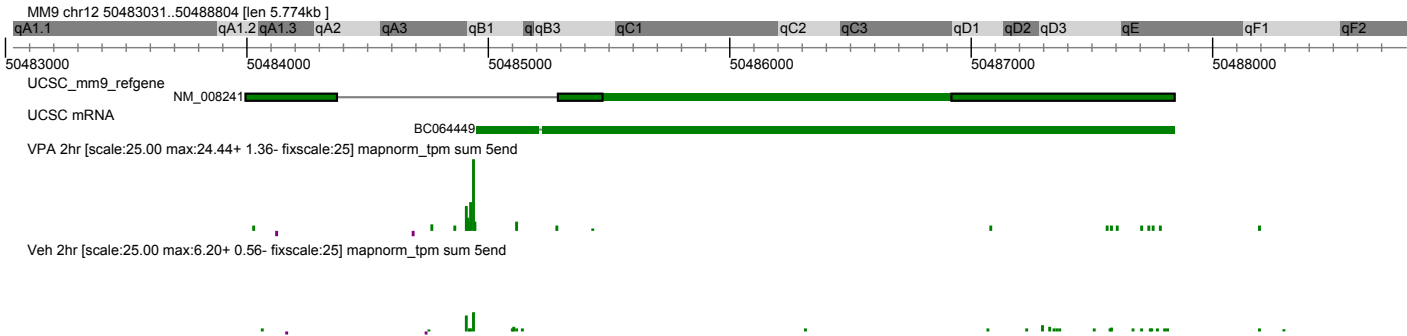

B

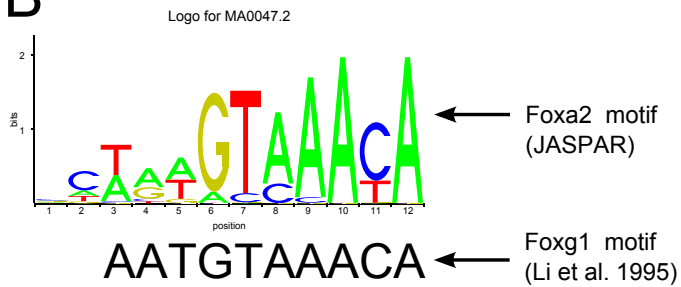

C

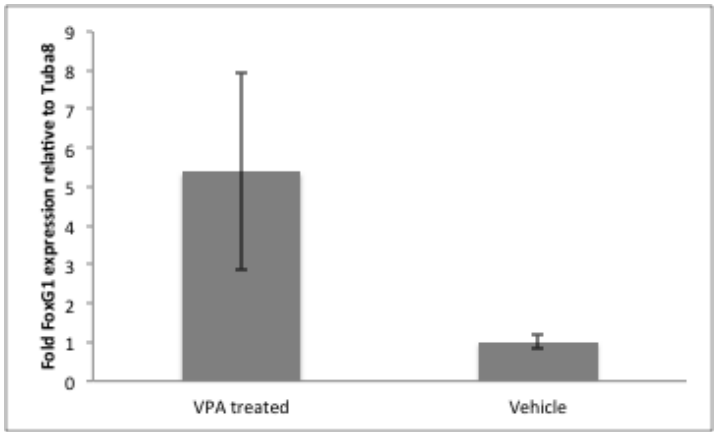

Supplement: Supplementary file 5 — 10.1186/s13072-015-0043-3 VPA-induced FoxG1 expression with binding sites which overlap VPA-induced DHSS. (A) Genome browser view for the Foxg1 locus. CAGE tags are significantly upregulated in the VPA sample compared to vehicle, at a start site downstream of the canonical RefSeq TSS, corresponding to previously described transcript BC064449. (B) Comparison between the Foxg1 binding motif and the Foxa2 motif. (C) qPCR validation for induced FoxG1 expression after 2 h VPA treatment. [file 13072_2015_43_MOESM5_ESM.pdf]
